# The Diagnosis of the Cause of Ascites

**Published:** 1903-07-18

**Authors:** 


### THE DIAGNOSIS OF THE CAUSE OF ASCITES.

Although it is not always an easy and straightforward matter to diagnose the existence of an effusion into the peritoneal cavity, yet it is more difficult, as a rule, to be quite sure about the cause of ascites. In a clinical lecture, published in the *Guy's Hospital Gazette*, Dr. L. E. Shaw points out that cases may be roughly classified into three groups:—1. Ascites which is associated with general dropsy. 2. Ascites due to portal obstruction. 3. Effusion due to some pathological condition of the peritoneum. Ascites which is part of a general anasarca is commonly due to heart failure or to an altered state of the blood such as occurs in chronic renal disease. It is to be remembered that although it is common to speak of general dropsy, yet it is rare for the condition to be equally distributed throughout the body, and the disproportion may occur so that the peritoneal effusion is in great excess and a difficulty at once arises in diagnosing the cause. In such a case the heart may be beating irregularly and a bruit may be audible and yet the origin of the trouble may not be cardiac. In the same way the kidneys may be secondarily affected and therefore the presence of albuminuria is not sufficient to indicate that renal disease is the primary cause of the dropsy. In general anasarca due to Bright's disease there may be perihepatitis present leading to an amount of ascites out of all proportion to the general dropsy. If ascites is due to obstruction of the portal vein there may be other evidences also of portal obstruction such as enlargement of the spleen, hæmorrhoids, sickness and diarrhœa with possibly a history of hæmatemesis. The condition which causes pressure on the portal vein may give other evidence of its existence, for example, the liver may be enlarged or there may be jaundice. Moreover, the lesion producing the portal obstruction may be associated with other lesions having the same cause. If, for instance, portal obstruction is due to alcoholic cirrhosis of the liver, other signs of alcoholism may be apparent elsewhere—in the patient's complexion, in his mind, and in his nervous system generally. Finally, if ascites is due to some pathological condition of the peritoneum itself it is likely that some evidence of this will be forthcoming. There will probably be adhesions between the various parts of the peritoneum, there may deposits in the omentum or glands, and there is likely to ensue a shrinking of the omentum and the mesenteries. If adhesions are present they may cause the fluid to be loculated, and this will lead to an absence of the free interchange of dulness and resonance as the posture of the patient is varied. Another point is that if adhesions are present there may not be the perfect mobility of the intestines which causes the normal abdomen to feel quite uniform wherever it is pressed. If the mesentery is shrunk, or if the intestines are bound down by adhesions, there will be no area of resonance in the front of the abdomen such as is usually found in cases of ascites. The chief diseases of the peritoneum which lead to ascites are (1) tubercle, (2) cancer, and (3) simple chronic peritonitis. The first two of which will generally be recognised owing to the patient's age, the presence or absence of cachexia, the course of the temperature, and other signs of tubercle or malignant disease in the body. Simple chronic peritonitis is a condition that is not clearly defined, and it may be that many of the cases so-called are really instances of tubercle of the peritoneum. In any case the diagnosis can only be arrived at by a process of exclusion. Dr. Shaw draws attention to the fact that ascites may be the cause of chronic thickening of the peritoneum as well as the result of it, and therefore the finding of a thickened opaque peritoneum in association with ascites does not always warrant the conclusion that the effusion is the result of chronic inflammation.

